# Pediatric contrast-enhanced ultrasound in clinical practice: evidence maturity and workflow integration

**DOI:** 10.3389/fradi.2026.1905765

**Published:** 2026-09-23

**Authors:** Han Yao, Xuejiao Li, Zhao Na, Xin Yi, Chenghao Zhanghuang

**Affiliations:** 1 Department of Ultrasound, Kunming Children’s Hospital (Children’s Hospital Affiliated to Kunming Medical University), Kunming, China; 2 Department of General Surgery, Kunming Children’s Hospital (Children’s Hospital Affiliated to Kunming Medical University), Kunming, China; 3 Department of Urology, Kunming Children’s Hospital (Children’s Hospital Affiliated to Kunming Medical University), Kunming, China; 4 Yunnan Province Clinical Research Center for Children’s Health and Disease; Yunnan Key Laboratory of Children’s Major Disease Research, Kunming Children's Hospital, Kunming, China

**Keywords:** children, contrast-enhanced ultrasound, contrast-enhanced voiding urosonography, evidence maturity, pediatric imaging, radiation-free imaging, vesicoureteral reflux, workflow integration

## Abstract

Contrast-enhanced ultrasound (CEUS) has become an important adjunct in pediatric imaging because it enables real-time assessment of microvascular perfusion without ionizing radiation and without nephrotoxic contrast media. This narrative review synthesizes current evidence and practical applications of pediatric CEUS, with emphasis on intravenous blood-pool CEUS, contrast-enhanced voiding urosonography (ceVUS), and selected luminal or hydrosonographic gastrointestinal applications. Literature was identified through targeted searches of PubMed/MEDLINE and Google Scholar, with priority given to pediatric-specific reviews, systematic reviews, consensus or implementation papers, and key original studies, supplemented by recent publications through March 2026. Current evidence is strongest for ceVUS, focal liver lesion characterization, and targeted CEUS for hemodynamically stable blunt abdominal trauma; in contrast, many non-hepatic abdominal, bowel, interventional, emergency, and critical-care applications remain center-dependent or exploratory and require more standardized pediatric validation. Among pediatric applications, ceVUS remains the most mature and reproducible, offering high diagnostic performance without radiation while also allowing detection of intrarenal reflux. However, evidence maturity differs substantially across indications, and regulatory heterogeneity, operator dependence, reimbursement barriers, differences in technology availability, professional training requirements, limited cost-effectiveness data, and the need for standardized protocols remain important implementation challenges. This review proposes an indication-specific evidence-maturity framework that distinguishes mature applications suitable for broader adoption from center-dependent applications requiring local expertise and local protocolization, and from emerging applications requiring stronger prospective validation. The framework is intended as a pragmatic, non-validated clinical organizing tool rather than as a formal evidence-grading system. Overall, CEUS is well positioned to expand within child-centered imaging pathways that seek to maximize diagnostic yield while minimizing radiation exposure, sedation, and procedural burden.

## 1 Introduction

Ultrasound is the cornerstone of many pediatric imaging pathways, but conventional sonography can be limited when lesion vascularity or tissue perfusion must be assessed in real time. Computed tomography (CT) and contrast-enhanced magnetic resonance imaging (MRI) provide complementary information, yet their use in children is constrained by ionizing radiation, sedation requirements in selected age groups, contrast-related concerns, cost, and reduced bedside availability (1–4). Against this background, contrast-enhanced ultrasound (CEUS) has emerged as a particularly attractive technique because it provides dynamic visualization of microvascular flow while avoiding ionizing radiation and nephrotoxic contrast exposure (1–4).

Pediatric CEUS now extends well beyond focal liver lesion characterization. The scope includes intravenous blood-pool CEUS, intracavitary microbubble applications such as ceVUS, and selected saline-assisted hydrosonographic gastrointestinal techniques. These approaches should be distinguished because they differ in route of administration, contrast preparation, procedural requirements, image-acquisition protocols, and safety considerations. Contemporary literature most consistently supports ceVUS for VUR evaluation, focal liver lesion characterization, and targeted CEUS for hemodynamically stable blunt abdominal trauma, whereas renal/adrenal, splenic, pancreatic, gallbladder, bowel, interventional, emergency, and critical-care applications have expanded but remain heterogeneous in evidence maturity and standardization (4–18, 20, 21). Recent practical and emergency-focused reviews have also reinforced selected CEUS roles in acute pediatric care and structured clinical imaging pathways (20, 21). Foundational ceVUS safety evidence and selected hydrosonographic gastrointestinal studies remain important for specific urinary and gastrointestinal applications (22, 23).

Nevertheless, the expanding application spectrum has created an important interpretive challenge: not all pediatric CEUS indications are supported by the same level of evidence, technical reproducibility, regulatory clarity, or workflow readiness. A practical framework is therefore needed to separate mature indications that can be incorporated into routine pathways from applications that should remain targeted, center-dependent, or investigational.

This review provides a clinically oriented synthesis of pediatric CEUS with three objectives: to summarize the current evidence for major pediatric applications, to propose an indication-specific evidence-maturity framework, and to identify practical steps for workflow integration. The proposed framework is summarized in Figure 1.

**Figure 1:**
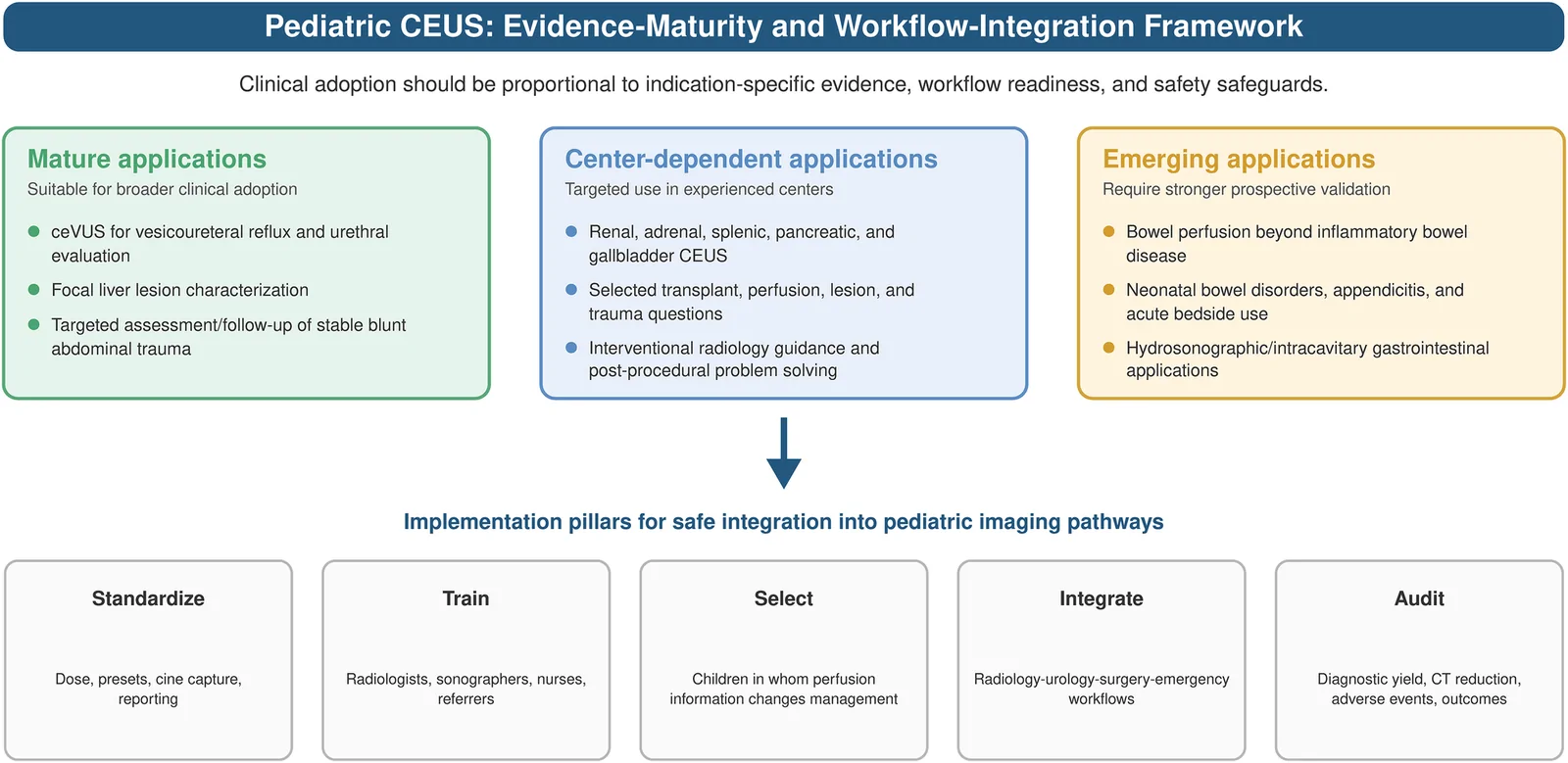
Proposed evidence-maturity and workflow-integration framework for pediatric contrast-enhanced ultrasound. The figure stratifies pediatric CEUS applications into mature, center-dependent, and emerging categories, and highlights implementation pillars that support safe integration into child-centered imaging pathways. CEUS, contrast-enhanced ultrasound; ceVUS, contrast-enhanced voiding urosonography; CT, computed tomography.

## 2 Literature search strategy

This article was prepared as a narrative review intended for clinical practice rather than a formal systematic review or meta-analysis. Targeted literature searches were performed in PubMed/MEDLINE and Google Scholar for English-language publications from January 2019 through March 2026 using combinations of the terms “contrast-enhanced ultrasound,” “CEUS,” “children,” “pediatric,” “blunt abdominal trauma,” “focal liver lesion,” “bowel,” “interventional radiology,” “contrast-enhanced voiding urosonography,” “ceVUS,” “vesicoureteral reflux,” and “Hirschsprung disease.” Priority was given to pediatric-specific reviews, systematic reviews, consensus or implementation papers, and key original studies. Earlier landmark studies were retained selectively when they remained directly relevant to foundational safety or validation data, including the large prospective ceVUS safety series that remains a key source of intravesical contrast-safety evidence (22).

Because the objective was a practical synthesis, results are presented according to clinical applicability, evidence maturity, reproducibility, and workflow relevance rather than by prespecified quantitative pooling. The review protocol was not registered, study selection was not performed using a reproducible systematic-review process, and no formal methodological quality or risk-of-bias assessment was performed. The conclusions should therefore be interpreted as a clinically oriented state-of-the-art synthesis rather than as pooled evidence or formal guideline recommendations.

## 3 Technical principles, safety, and implementation considerations

CEUS uses gas-filled microbubbles that remain confined to the vascular or luminal space and generate strong nonlinear harmonic signals when insonated at a low mechanical index. In children, examination quality depends on appropriate scanner presets, use of contrast-specific imaging modes, careful timing, weight-adjusted dosing, and disciplined cine acquisition (1). For intravenous pediatric CEUS, sulfur hexafluoride microbubbles are commonly administered using weight-based protocols, often around 0.03 mL/kg, followed by a saline flush and continuous low-mechanical-index imaging of the region of interest (1, 4, 20).

Available pediatric safety data are reassuring. A comprehensive pediatric safety review reported a very low frequency of adverse events, most of which were mild and self-limited (2). Intravesical administration for ceVUS also has a strong safety record, with minor events usually related to catheterization rather than the contrast agent itself (22). Nevertheless, regulatory approval, age-specific labeling, and reimbursement remain heterogeneous across countries, and several pediatric indications are still used off label despite growing published experience (3, 4).

From an implementation standpoint, CEUS is especially attractive in children because it is portable, repeatable, and frequently feasible without sedation. These strengths are most relevant when serial perfusion assessment is desirable, when CT avoidance is a priority, or when bedside problem solving is needed (1, 2, 4, 20). However, implementation should not be reduced to availability of an ultrasound scanner alone. Safe integration requires protocolized patient selection, staff training, contrast safety preparation, structured image acquisition, clear reporting language, and institutional planning for contrast-agent access, billing or reimbursement, and quality assurance. Key practical considerations for pediatric CEUS implementation are summarized in Table 1.

**Table 1:** Practical considerations for implementation of pediatric CEUS.

Domain	Key points	Practical implications
Patient selection	Most useful when perfusion information will change management and when radiation avoidance is desirable.	Particularly relevant in children requiring follow-up or bedside reassessment.
Preparation	Age-appropriate explanation, IV access or bladder catheterization when needed, and coordination between radiologist, technologist, nurse, and referring clinician.	Good preparation reduces motion, repeat injections, and procedural stress.
Technique	Use contrast-specific low-mechanical-index presets, weight-adjusted dosing, continuous cine capture, and timely review of arterial and late phases.	Protocol discipline is essential for reproducible examinations and confident interpretation.
Safety	Published pediatric safety data are reassuring for both intravenous CEUS and ceVUS.	Emergency drugs and trained staff should still be available as part of standard contrast safety practice.
Workflow	CEUS is portable and frequently feasible without sedation.	Valuable for rapid problem solving, serial follow-up, and CT reduction strategies.
Interpretation	Findings must be integrated with clinical context, laboratory data, and when required other imaging modalities.	CEUS should complement rather than replace sound clinical decision making.
Limitations	Operator dependence, limited whole-body coverage, heterogeneous regulatory approval, reimbursement uncertainty, technology availability, and limited pediatric cost-effectiveness data remain important barriers.	Local training, protocol standardization, indication selection, and local audit of feasibility, safety, and downstream resource use are critical for wider adoption.
System-level barriers	Regulatory labeling, contrast-agent access, reimbursement, equipment capability, workforce training, and cost-effectiveness may differ substantially across institutions and countries.	Implementation plans should be adapted to local approval status, billing pathways, equipment resources, staff competency, and institutional quality-assurance capacity.

## 4 Intravenous CEUS applications in pediatric practice

Intravenous or blood-pool CEUS represents the broadest category of pediatric CEUS applications. It should generally be viewed as a complementary technique that improves diagnostic confidence, reduces CT utilization in selected scenarios, and refines management when grayscale or Doppler ultrasound is inconclusive. Major pediatric applications, strengths, limitations, and comparator modalities are summarized in Table 2.

**Table 2:** Major pediatric CEUS applications, strengths, and practical limitations.

Application	Typical role	Major strengths	Main limitations/cautions	Comparator modalities
Blunt abdominal trauma	Targeted assessment and follow-up of solid-organ injury in hemodynamically stable children	Radiation-free, repeatable, sensitive for parenchymal perfusion defects	Does not replace whole-body trauma CT; limited for retroperitoneal, thoracic, and osseous injury	CT; conventional ultrasound
Focal liver lesions	Characterization of benign vs. malignant lesions and follow-up of indeterminate findings	Dynamic enhancement patterns, no nephrotoxic contrast, strong pediatric evidence base	Interpretation must consider age, tumor markers, and clinical context	MRI; CT
Kidney/adrenal/spleen/pancreas/gallbladder	Problem solving for perfusion abnormalities, focal lesions, trauma, or transplant-related questions	Adds vascular and perfusion detail beyond grayscale and Doppler ultrasound	Less standardized and more operator-dependent than liver CEUS or ceVUS; prospective pediatric outcome data and standardized diagnostic criteria remain limited. Center-dependent; not evidence-equivalent to ceVUS, liver CEUS, or stable trauma CEUS.	CT; MRI; conventional ultrasound
Bowel perfusion/inflammatory bowel disease	Assessment of bowel wall and mesenteric perfusion and treatment monitoring	Potential for real-time inflammatory activity assessment	Evidence is strongest in inflammatory bowel disease; neonatal bowel disorders, appendicitis, ICU/ED perfusion use, and broader indications remain emerging Routine first-line use outside IBD is not yet standardized.	MRI enterography; conventional intestinal ultrasound
Interventional radiology	Biopsy guidance, drain assessment, viability mapping, and post-procedural problem solving	Improves targeting and may reduce CT-guided procedures	Requires operator expertise, technology availability, and workflow integration Center-dependent; local audit and comparator imaging are advisable.	CT-guided or conventional ultrasound-guided procedures
ceVUS for VUR	Detection and grading of reflux and urethral evaluation	Radiation-free, dynamic, high diagnostic performance, detects intrarenal reflux	Requires catheterization and protocol standardization	Voiding cystourethrogram; radionuclide cystography
Hydrosonographic gastrointestinal applications	Selected evaluation of Hirschsprung disease and congenital/obstructive bowel disorders	Potential radiation-free adjunct in selected centers	Evidence is promising but less mature; protocols are not standardized and the technique is not interchangeable with intravenous CEUS	Contrast enema; conventional ultrasound

### 4.1 Blunt abdominal trauma

Blunt abdominal trauma is one of the best-established pediatric indications for CEUS. In hemodynamically stable children with isolated low- to moderate-energy abdominal trauma, CEUS can detect and monitor solid-organ injury while avoiding additional radiation exposure (5, 6). Traumatic lacerations, infarcts, and hematomas typically appear as focal or geographic nonenhancing regions within otherwise enhancing parenchyma, and pseudoaneurysms or active extravasation may also be demonstrated in selected cases (5).

Subsequent literature has reinforced this position while clarifying the technique's boundaries. CEUS is not a replacement for whole-body trauma CT, and it cannot by itself exclude retroperitoneal injury, thoracic injury, or osseous trauma (5, 6, 21). Accordingly, the most defensible role for CEUS is targeted evaluation and follow-up in hemodynamically stable children, especially when clinical concern focuses on abdominal solid-organ injury and repeated CT is undesirable.

### 4.2 Focal liver lesions

Focal liver lesion characterization remains the most established intravenous CEUS indication in pediatric imaging. Dynamic arterial, portal venous, and late-phase enhancement patterns improve diagnostic confidence in differentiating focal nodular hyperplasia, hemangioma, hepatoblastoma, and other benign or malignant lesions (7). CEUS is particularly useful when grayscale and Doppler findings are indeterminate and when radiation-free lesion characterization is desirable (7, 8).

Pediatric-specific interpretive work has moved beyond simple extrapolation of adult frameworks. A pilot pediatric liver CEUS criterion proposed by Wang et al. improved differentiation of benign and malignant focal liver lesions, particularly hepatoblastoma vs. hemangioma (8). CEUS has also shown value in surveillance of liver lesions identified after treatment of childhood malignancies, where serial follow-up without repeated CT is advantageous (9). Even so, CEUS findings should be interpreted together with age, alpha-fetoprotein level, oncologic history, underlying liver disease, and when necessary cross-sectional imaging or tissue diagnosis (7–9).

### 4.3 Kidney, adrenal, splenic, pancreatic, and gallbladder applications

Pediatric CEUS use outside the liver is expanding steadily. Reported applications include renal perfusion abnormalities, focal renal lesions, complex cystic lesions, transplant complications, selected adrenal abnormalities, splenic trauma, pancreatic perfusion, and gallbladder pathology (10, 11). These are clinically appealing scenarios because conventional ultrasound may be insensitive while CT may be undesirable or unnecessary.

However, nonhepatic applications remain more dependent on operator experience and institutional familiarity than liver CEUS or ceVUS. The evidence base is encouraging but comparatively less robust, prospective pediatric outcome studies remain limited, and standardized diagnostic thresholds are not yet available for many renal, adrenal, splenic, pancreatic, and gallbladder scenarios (10, 11). In daily practice, these examinations are best framed as targeted problem-solving studies performed in experienced centers rather than universally standardized first-line tests.

### 4.4 Bowel perfusion and inflammatory bowel disease

In the pediatric bowel literature, the strongest intravenous CEUS evidence concerns bowel wall and mesenteric perfusion rather than classic intracavitary contrast studies. CEUS can help assess inflammatory activity in inflammatory bowel disease, especially Crohn disease, and may support treatment monitoring through qualitative enhancement patterns and quantitative contrast kinetics (12).

At the same time, evidence outside inflammatory bowel disease is less mature. Applications in necrotizing enterocolitis, appendicitis, neonatal bowel disorders, intensive care, and emergency bedside perfusion assessment remain promising but insufficiently standardized for broad routine adoption (12, 19, 21). Outside inflammatory bowel disease, bowel CEUS should be regarded as emerging or locally audited rather than a routine first-line pathway, and its evidence maturity should not be equated with ceVUS, focal liver CEUS, or stable trauma CEUS. It is therefore important not to overstate the maturity of gastrointestinal CEUS by conflating intravenous bowel perfusion imaging with hydrosonographic or saline-assisted luminal techniques.

### 4.5 Interventional and bedside applications

CEUS has become increasingly relevant in pediatric interventional radiology, where it can improve lesion targeting, delineate viable tissue, assess collections, confirm drainage pathways, and reduce reliance on CT guidance in selected procedures (13). These advantages are particularly attractive in children because they can shorten workflows and reduce cumulative procedural burden.

The same portability and repeatability also make CEUS valuable as a bedside problem-solving tool. Current evidence is strongest for abdominal trauma and selected perfusion questions, whereas wider emergency and critical care applications are still evolving (5, 6, 13, 21). Institutional workflow, machine capability, and operator training remain major determinants of successful implementation. These bedside and interventional uses should therefore be framed as adjunctive, center-dependent problem-solving applications rather than uniformly mature clinical pathways.

## 5 Contrast-enhanced voiding urosonography

Among all pediatric CEUS applications, ceVUS is arguably the most mature. Its principal advantage is that it enables dynamic imaging of the urinary tract without ionizing radiation, which is particularly important in children with recurrent urinary tract infection, suspected reflux nephropathy, or anticipated follow-up imaging (14–16).

### 5.1 Vesicoureteral reflux detection and grading

ceVUS is a well-established and sensitive method for detecting and grading VUR (14). Modern protocols can also provide clinically meaningful urethral assessment, particularly in boys, thereby addressing one of the historic criticisms of ultrasound-based reflux imaging (15).

Comparative evidence is favorable. A systematic review and meta-analysis reported excellent overall diagnostic performance of ceVUS relative to radiographic standards, supporting its use as a radiation-free alternative in appropriately selected children (16). In practice, the greatest value of ceVUS is achieved when centers apply standardized catheterization, contrast preparation, bladder filling, and image acquisition protocols.

### 5.2 Intrarenal reflux and prognostic implications

A major strength of ceVUS is its ability to detect intrarenal reflux. This is clinically important because intrarenal reflux is increasingly recognized as more than a technical curiosity and may identify a subgroup at increased risk for renal parenchymal injury (17, 18).

In children with recurrent febrile urinary tract infection, high-grade reflux, or concern for reflux nephropathy, identification of intrarenal reflux can add clinically relevant risk-stratification information beyond a simple present-or-absent VUR classification (17, 18). This feature further strengthens the role of ceVUS in child-centered urinary imaging pathways.

### 5.3 Safety and implementation considerations

Large pediatric experience supports the safety of intravesical contrast administration. In a prospective series of 1,010 children undergoing ceVUS with a second-generation ultrasound contrast agent, no serious adverse events were reported, and the few minor events were considered more likely related to catheterization than to the contrast agent itself (22).

Taken together, current literature supports ceVUS as one of the clearest examples of how CEUS can replace an ionizing-radiation examination in children without compromising diagnostic performance (14–18, 22). Wider adoption depends less on proof of principle and more on local expertise, workflow design, and collaboration between pediatric radiology and pediatric urology teams.

## 6 Hydrosonographic and intracavitary gastrointestinal applications

The gastrointestinal CEUS literature in children requires careful wording. The strongest evidence for congenital and obstructive bowel conditions comes from saline-assisted or hydrosonographic techniques rather than from standard intraluminal microbubble CEUS. In a prospective study, hydrocolonic sonography showed high sensitivity and specificity for Hirschsprung disease and correlated well with contrast enema findings, suggesting a potential radiation-free screening role in selected children (23).

Broader sonographic work in neonatal bowel obstruction likewise supports a growing role for ultrasound in congenital and obstructive gastrointestinal disease, although that literature is heterogeneous and not CEUS-specific (19). Accordingly, gastrointestinal intracavitary applications are best described as promising and evolving rather than as fully established CEUS indications on par with trauma CEUS or ceVUS.

## 7 Discussion: evidence maturity and clinical workflow integration

A practical pediatric CEUS strategy should be indication-specific rather than technology-driven. The key question is not whether CEUS is technically feasible, but whether it provides actionable information that changes management, reduces higher-burden imaging, improves patient safety, or supports longitudinal follow-up. For the purposes of this narrative synthesis, pediatric CEUS applications are stratified into mature, center-dependent, and emerging categories (Table 3). This categorization is based on the authors' qualitative assessment of pediatric evidence consistency, protocol reproducibility, availability of clinical comparators, regulatory and workflow readiness, and likely management impact. It should be understood as a pragmatic interpretive framework rather than a formally validated grading system.

**Table 3:** Proposed evidence-maturity and workflow-integration framework for pediatric CEUS.

Evidence-maturity category	Representative pediatric applications	Suggested clinical integration	Key safeguards/research needs
Mature	ceVUS for VUR and urethral assessment; focal liver lesion characterization; targeted CEUS for stable blunt abdominal trauma.	Eligible for structured institutional pathways when trained personnel and contrast safety procedures are available.	Standardized protocols, structured reporting, adverse-event documentation, and periodic audit of diagnostic yield and CT reduction.
Center-dependent	Renal, adrenal, splenic, pancreatic, gallbladder, transplant-related and selected interventional radiology applications.	Use as targeted problem-solving studies in experienced centers, ideally with explicit clinical questions, local protocols, and comparator imaging when available.	Local training, senior supervision during implementation, technology and contrast-agent availability, outcome review, and cautious interpretation outside validated indications.
Emerging	Bowel perfusion beyond inflammatory bowel disease; neonatal bowel disorders; appendicitis; broad urgent/emergent bedside and intensive care use; selected hydrosonographic or intracavitary gastrointestinal applications.	Prefer research protocols, prospective registries, or carefully audited local pathways rather than routine first-line adoption.	Prospective validation, reproducible quantitative or qualitative criteria, management-impact studies, patient-centered outcome assessment, and cost-effectiveness evaluation. Include resource-utilization and cost-effectiveness endpoints when CEUS is used to reduce CT, MRI, sedation, or repeat imaging.

### 7.1 Mature applications suitable for broader clinical adoption

Mature applications are those supported by relatively consistent pediatric evidence, reproducible examination protocols, and clear clinical comparators. ceVUS is the leading example because it provides dynamic radiation-free evaluation of reflux, grading, urethral assessment, and intrarenal reflux (14–18, 22). Focal liver lesion characterization and CEUS in hemodynamically stable blunt abdominal trauma also have strong practical footing because they address clinically common questions in which radiation avoidance and real-time perfusion assessment are particularly valuable (5–9).

For these applications, implementation can reasonably move from individual expert use toward structured institutional pathways. Examples include defined indications for ceVUS after recurrent urinary tract infection, CEUS algorithms for indeterminate pediatric liver lesions, and trauma protocols that use CEUS for targeted follow-up of known or suspected solid-organ injury in stable children.

### 7.2 Center-dependent applications requiring local expertise and protocol standardization

Center-dependent applications are clinically useful but more dependent on local expertise, equipment, and interdisciplinary workflow than mature applications. This category includes many renal, adrenal, splenic, pancreatic, gallbladder, transplant-related, and interventional radiology applications (10, 11, 13). CEUS may provide decisive information in selected patients, but protocols, reporting language, and interpretation criteria are less standardized than for ceVUS or liver CEUS.

For these indications, CEUS should be introduced through targeted pathways in experienced centers. A defensible workflow includes explicit clinical questions, senior radiologist supervision during early implementation, documentation of comparator imaging when available, technology and contrast-agent availability, and periodic review of diagnostic yield and downstream management changes.

### 7.3 Emerging applications requiring stronger prospective validation

Emerging applications include areas where feasibility is promising but routine use remains constrained by limited pediatric validation, variable protocols, or uncertain outcome impact. Examples include bowel perfusion imaging beyond inflammatory bowel disease, neonatal bowel disorders, appendicitis, broad emergency and critical care indications, intensive care applications, and selected hydrosonographic or intracavitary gastrointestinal applications (12, 19, 21, 23). These areas should not be described as having the same evidence maturity as ceVUS, liver CEUS, or stable trauma CEUS until prospective pediatric outcome data and standardized diagnostic criteria are available.

These applications should not be dismissed, but they should be framed appropriately. At present, they are best suited to research protocols, prospective registries, or carefully audited institutional pathways. Future studies should focus not only on diagnostic accuracy but also on whether CEUS changes management, reduces CT or sedation, shortens time to decision, improves follow-up, or affects patient-centered outcomes.

### 7.4 Implementation barriers and actionable recommendations

Several barriers must be addressed before pediatric CEUS can be more uniformly embedded into routine imaging pathways. Regulatory status also differs by jurisdiction and indication. For example, in the United States, FDA labeling for Lumason includes selected pediatric intravenous and intravesical applications, including focal liver lesion characterization and evaluation of vesicoureteral reflux, whereas the European labeling environment for SonoVue has historically been more indication- and route-specific, with pediatric approval particularly established for urinary tract assessment. Consequently, many other pediatric intravenous abdominal and bedside applications remain off label or locally governed despite increasing clinical evidence. Regulatory approval and reimbursement remain inconsistent, especially for pediatric and off-label indications (3, 4). Technology availability also varies across centers, because high-quality CEUS requires contrast-specific software, appropriate low-mechanical-index presets, reliable contrast-agent supply, image-storage capacity for cine loops, and personnel familiar with pediatric dosing and safety procedures. Operator dependence is unavoidable, and examination quality may vary substantially with scanner settings, contrast dosing, patient cooperation, cine acquisition, and reporting experience (1, 2, 4). Although pediatric cost-effectiveness evidence remains limited, a single-center retrospective analysis suggested that pediatric CEUS may reduce subsequent CT/MRI use and imaging-related resource utilization, supporting its potential cost-effectiveness in selected institutional pathways (24). These findings should be interpreted as institution-specific until broader multicenter pediatric cost-effectiveness and workflow-impact data are available.

Actionable recommendations include: (1) develop indication-specific protocols for dosing, presets, acquisition timing, and reporting; (2) maintain emergency preparedness and contrast safety documentation; (3) train radiologists, sonographers, nurses, and referring clinicians together; (4) define clinical pathways in collaboration with pediatric surgery, emergency medicine, oncology, nephrology, urology, and intensive care teams where appropriate; (5) use structured reporting language that states both the CEUS finding and the limitations of the examination; (6) document local reimbursement, contrast availability, and technology requirements before broad implementation; and (7) audit diagnostic yield, adverse events, CT reduction, repeat imaging, downstream clinical decisions, and, where feasible, cost-related outcomes.

This evidence-maturity approach may help avoid two common errors: overextending CEUS into unsupported indications and underusing it where evidence already supports meaningful clinical benefit. The goal is not to replace CT, MRI, or conventional ultrasound indiscriminately, but to position CEUS where its unique combination of real-time perfusion imaging, repeatability, bedside feasibility, and radiation avoidance provides the greatest pediatric value.

### 7.5 Limitations of this review and framework

Several limitations should be acknowledged. First, this article is a narrative review rather than a systematic review or meta-analysis. Although the literature search was targeted and clinically oriented, it was not designed to be fully reproducible, did not include a registered protocol, and did not include formal assessment of methodological quality or risk of bias. The review therefore cannot determine pooled diagnostic accuracy, comparative effectiveness, or the overall certainty of evidence for each indication.

Second, the proposed evidence-maturity framework has not been formally validated and relies on author interpretation. The terms mature, center-dependent, and emerging are intended to describe the combined status of evidence strength, protocol reproducibility, workflow readiness, and clinical adoption rather than to function as rigid guideline categories. Third, evidence is heterogeneous across pediatric CEUS applications. ceVUS, focal liver lesion characterization, and targeted CEUS for stable blunt abdominal trauma are supported by comparatively stronger data and more standardized protocols, whereas many nonhepatic organ applications, bowel perfusion indications, neonatal conditions, appendicitis, emergency and intensive care uses, and hydrosonographic gastrointestinal applications still require prospective pediatric validation and standardized diagnostic criteria. These limitations constrain generalizability and should temper universal adoption outside experienced centers.

## 8 Conclusion

CEUS has evolved into a clinically meaningful component of pediatric imaging rather than a niche adjunctive technique. Current evidence is most mature for ceVUS for VUR evaluation, focal liver lesion characterization, and targeted assessment or follow-up of hemodynamically stable blunt abdominal trauma. Selected renal and other non-hepatic abdominal applications, bowel perfusion assessment, interventional radiology, and emergency/ICU uses are clinically promising but should generally be considered center-dependent or emerging applications rather than evidence-equivalent routine indications.

Its principal advantages in children are the absence of ionizing radiation, real-time perfusion imaging, repeatability, and bedside feasibility. However, not all pediatric applications are equally mature, and hydrosonographic gastrointestinal techniques should not be presented as interchangeable with intravenous CEUS or ceVUS. A pragmatic, indication-specific evidence-maturity framework can support more responsible adoption by distinguishing mature applications suitable for broader clinical integration from center-dependent applications that require local expertise and protocol standardization, and from emerging applications that require prospective validation. With continued standardization and stronger outcome-oriented and cost-effectiveness studies, CEUS is likely to assume a larger role in child-centered imaging pathways designed to maximize diagnostic value while minimizing harm.
